# Construction of Machine Learning Models to Predict Changes in Immune Function Using Clinical Monitoring Indices in HIV/AIDS Patients After 9.9-Years of Antiretroviral Therapy in Yunnan, China

**DOI:** 10.3389/fcimb.2022.867737

**Published:** 2022-05-12

**Authors:** Bingxiang Li, Mingyu Li, Yu Song, Xiaoning Lu, Dajin Liu, Chenglu He, Ruixian Zhang, Xinrui Wan, Renning Zhang, Ming Sun, Yi-Qun Kuang, Ya Li

**Affiliations:** ^1^ Institute of Medical Biology, Chinese Academy of Medical Sciences and Peking Union Medical College, Kunming, China; ^2^ Department of Laboratory Medicine, Yunnan Provincial Institute of Infectious Diseases, Kunming, China; ^3^ Department of Laboratory Medicine, First Affiliated Hospital of Kunming Medical University, Kunming, China; ^4^ Yunnan Key Laboratory of Laboratory Medicine, First Affiliated Hospital of Kunming Medical University, Kunming, China; ^5^ Yunnan Innovation Team of Clinical Laboratory and Diagnosis, First Affiliated Hospital of Kunming Medical University, Kunming, China; ^6^ Department of Gynecology, First Affiliated Hospital of Kunming Medical University, Kunming, China; ^7^ Department of Medical Records and Statistics, First Affiliated Hospital of Kunming Medical University, Kunming, China; ^8^ Department of Disease Control and Prevention, The First People’s Hospital of Yunnan Province, The Affiliated Hospital of Kunming University of Science and Technology, Kunming, China; ^9^ National Health Commission (NHC) Key Laboratory of Drug Addiction Medicine, First Affiliated Hospital of Kunming Medical University, Kunming Medical University, Kunming, China; ^10^ Scientific Research Laboratory Center, First Affiliated Hospital of Kunming Medical University, Kunming, China

**Keywords:** HIV/AIDS, RF, MLP, SVM, machine learning model, CD4/CD8 ratio, prediction, ART

## Abstract

**Objective:**

To investigate trends in clinical monitoring indices in HIV/AIDS patients receiving antiretroviral therapy (ART) at baseline and after treatment in Yunnan Province, China and to provide the basis for guiding clinical treatment to obtain superior clinical outcomes.

**Methods:**

A total of 96 HIV/AIDS patients who had started and persisted in highly active ART treatment from September 2009 to September 2019 were selected. Of these, 54 had a CD4 cell count < 200 cells/μl while 42 had a CD4 cell count ≥ 200 cells/μl. Routine blood tests, liver and renal function, and lipid levels were measured before and 3, 6, 9, and 12 months after treatment. Lymphocyte subset counts and viral load were measured once per year, and recorded for analysis and evaluation. Three machine learning models (support vector machine [SVM], random forest [RF], and multi-layer perceptron [MLP]) were constructed that used the clinical indicators above as parameters. Baseline and follow-up results of routine blood and organ function tests were used to analyze and predict CD4^+^ T cell data after treatment during long-term follow-up. Predictions of the three models were preliminarily evaluated.

**Results:**

There were no statistical differences in gender, age, or HIV transmission route in either patient group. Married individuals were substantially more likely to have <200 CD4^+^ cells/μl. There was a strong positive correlation between ALT and AST (r = 0.587) and a positive correlation between CD4 cell count and platelet count (r = 0.347). Platelet count was negatively correlated with ALT (r = -0.229), AST (r = -0.251), and positively correlated with WBCs (r = 0.280). Compared with the CD4 cell count < 200 cells/μl group, all three machine learning models exhibited a better predictive capability than for patients with a CD4 cell count ≥ 200 cells/μl. Of all indicators, the three models best predicted the CD4/CD8 ratio, with results that were highly consistent. In patients with a CD4 cell count < 200 cells/μl, the SVM model had the best performance for predicting the CD4/CD8 ratio, while the CD4/CD8 ratio was best predicted by the RF model in patients with a CD4 cell count ≥ 200 cells/μl.

**Conclusion:**

By the incorporation of clinical indicators in SVM, RF, and MLP machine learning models, the immune function and recuperation of HIV/AIDS patients can be predicted and evaluated, thereby better guiding clinical treatment.

## Introduction

AIDS remains a serious public health problem in China (Xu et al., 2014; Chen et al., 2015; Chen et al., 2018). Yunnan province is located on China’s southwestern border with Vietnam, Myanmar, and Laos and has a large cross-border population. Yunnan is also close to the Golden Triangle, China’s largest drug-producing region (Jia et al., 2008; Li et al., 2010; Li et al., 2016). In 1989, the first outbreak of human immunodeficiency virus type 1 (HIV-1) among injecting drug users occurred in Dehong Prefecture, Yunnan (Jia et al., 2011). Since then, Yunnan has become the center of an HIV-1 epidemic in China and the country’s worst-hit region for AIDS (Wang et al., 2015; Li et al., 2016; Zhu et al., 2018). Studies show that nearly 25% of new HIV cases in China come from Yunnan (Xiao et al., 2007; Duan et al., 2008; Chow et al., 2013), of which 92.6% are caused by unprotected sex (Su et al., 2016; Li et al., 2017; Zhu et al., 2018).

HIV is a retrovirus that primarily infects CD4^+^ T lymphocytes, leading to a progressive decline in their number, gradually weakening the host’s immune system leading to acquired immune deficiency syndrome (AIDS). In untreated infected patients, the numbers of CD4 cells decline progressively (Février et al., 2011), and so the CD4 cell count has become an important indicator for the selection of treatment plans and measurement of the effectiveness of antiretroviral therapy (ART) (Gazzard and Moyle, 1998; Carpenter et al., 2000; Dybul et al., 2002). In addition, the number of CD4^+^ T lymphocytes is an important indicator by which to judge the progression of the disease and evaluate patient prognosis. After receiving antiviral treatment, patients infected with HIV undergo a period of immune reconstruction of variable duration. Absolute T cell count is the most reliable indicator of disease progression (Abbass and Lichtman, 2008).

In recent years, multiple mathematical models have been established in which HIV infection is related to CD4^+^ T cell number (Hou and Ma, 2009; Wang and Song, 2009; Li and Ye, 2010). The results of these mathematical models provide theoretical guidance and suggestions for the prevention and treatment of AIDS. As early as 1999, the researcher Perelson et al. (Perelson and Nelson, 1999) introduced a model of HIV infection incorporating CD4^+^ T cell number. There have been many subsequent models based on CD4^+^ T cells as the principal variable. For example, Chen et al., (2007) used TLC(T lymphocyte,TLC) to predict CD4^+^ T cell count. Singh et al. (Singh and Mars, 2010) used a support vector machine with mined data to predict changes in CD4 cell count in HIV infected patients using genome sequencing, current viral load, and the number of weeks of follow-up as predictive indicators. Jingquan et al. (Wang et al., 2006) divided the CD4^+^ T lymphocyte counts of patients into three categories depending on the total lymphocyte count using a decision tree, predicting CD4^+^ T lymphocyte counts of less than 350/μL. Such estimates of CD4^+^ T cell number trajectory are essential for public health models as they predict the course of HIV epidemics (Eshun-Wilson et al., 2012; Rowley, 2014; Ren et al., 2017). However, the majority of such models include only routine blood data as parameters, with a follow-up time of less than three years. No studies have incorporated routine blood tests, lymphoid subgroup count, viral load, liver function, renal function, and blood lipids in addition to other indicators into the modeling, representing the innovative feature, and value, of the present study. The model will assist in the prompt prediction of immune response and timely adoption of adjuvant therapy to improve patient immune function. Machine learning models have widespread applications in medical research, almost any data type can be used to build predictive models. Since the inconsistent prediction accuracy in different models, the prediction results shared by multiple models are more accurate (Huang et al., 2018; Renganathan, 2019; Blanchet et al., 2020). This study used three machine learning methods to build the predictive model.

In Yunnan province, a relatively underdeveloped frontier province, it is not feasible to count CD4 or other lymphocyte subsets because these parameters depend on a flow cytometry platform. For confirmed HIV/AIDS patients and others during follow-up, indicators such as routine blood and liver function tests, *etc.* are more readily available, thus, the present study aims to construct three different models based on different baseline levels of CD4, CD8, the CD4/CD8 ratio and other follow-up results, among newly diagnosed HIV/AIDS patients with a CD4 cell count < 200 cells/μl and CD4 cell count ≥ 200 cells/μl. The model can predict changes in immune function and thereby calculate the prognosis of HIV/AIDS patients, allowing an appropriate selection of clinical antiviral drugs. The model has the potential for considerable cost savings for diagnosis and follow-up. The benefits to infected patients are clear.

## Methods

### Ethics Approval

The research protocol used in the present study has been reviewed by the Ethics Committee of the First Affiliated Hospital of Kunming Medical University. Informed consent was obtained from all participants included in the study prior to enrollment, and all information and data were confirmed for analysis.

### Sample Collection

A retrospective study was conducted on HIV/AIDS diagnosis and follow-up patients from the First Affiliated Hospital of Kunming Medical University. Of the 96 patients, the longest follow-up time was 9.9 years while the shortest was 2.6 years, with a median duration of 5.9 years. All confirmed patients were screened and the presence of HIV antibody confirmed by standard methods, Western blot analysis, and nucleic acid testing as a measure of HIV viral load as a supplementary test, if necessary. All confirmed patients were diagnosed in accordance with the national technical specifications for HIV/AIDS Testing, 2020.Among 96 patients with HIV/AIDS, according to the Chinese AIDS diagnosis and treatment guidelines, the main treatment regimen was lamivudine + zidovudine + efavirenz (3TC+AZT+EFV) and lamivudine + Zidovudine + nevirapine (3TC+AZT+NVP), and the dosage was strictly in accordance with the guidelines,and in accordance with China’s AIDS diagnosis and treatment guidelines.

Of the 96 HIV/AIDS patients who began and adhered to highly active ART (HAART) during the 10 year period from September 2009 to September 2019, 54 had a CD4 cell count of < 200 cells/μl while 42 had a count of ≥200 cells/μl. In accordance with the requirements of the National information management standards for free antiviral therapy, routine blood tests, liver and kidney function, and blood lipid levels were followed up 3, 6, 9, and 12 months before and after treatment. Free lymphocyte subset counts and viral load tests were performed once per year. All test results were recorded for analysis and evaluation.

The treatment plan and medical inclusion criteria for patients were those stated in the “National Manual for Free HIV Antiviral Treatment (2nd edition)”. All patients signed the “Informed Consent for Free HIV antiviral treatment” document, allowing drugs to be provided free of charge by the state.

### Laboratory Testing

A 2ml sample of venous blood was collected from each HIV/AIDS patient on an empty stomach at each time point. Blood cells were analyzed by flow cytometry (FACSCan II, BD Biosciences, San Jose, CA) using a combined CD3/CD4/CD8/CD45 Multitest reagent (BD Biosciences, San Jose, CA), allowing the absolute number of lymphocyte subsets to be measured and analyzed. All tests were completed less than 4 hours after venous blood collection. White blood cell (WBC) and platelet counts and hemoglobin (Hb) concentration were measured by routine blood testing using a Nisen Meikang automatic hematocyte counter (Japan). Total cholesterol (TC), total triglyceride (TG), alanine transaminase (ALT), aspartate aminotransferase (AST), and creatinine levels, aspects of blood lipids, and liver and renal function tests were performed using a Roche Cobas 8000 analyzer. Samples were prepared using a High Pure System Viral Acid kit, while a COBAS TaqMan 48 analyzer was used for automatic amplification and measurement. Samples were tested after routine daily indoor quality control testing.

### Data Preprocessing

For each sample, we deleted records with missing CD4/CD8 ratio or anti-HIV treatment, all variables (including gender, age, marital status, route of infection, liver and kidney function, blood lipid levels, routine blood tests, lymphoid subsets, and HIV viral load data at diagnosis and at each follow-up) were first normalized then processed in accordance with the following formulae:


$$x_{i}=v_{0}+\left[{\left(\frac{v_{1}-v_{0}}{d_{1}}\right)}^{2},{\left(\frac{v_{2}-v_{1}}{d_{2}-d_{1}}\right)}^{2},\ldots ,{\left(\frac{v_{i}-v_{i-1}}{d_{i}-d_{i-1}}\right)}^{2}\right]$$

$$y_{i}=\frac{d_{i+1}}{d_{i}}\cdot v_{i+1}$$

$$X=\left[\begin{array}{c} x_{1}\\ x_{2}\\ \vdots \\ x_{n} \end{array}\right],Y=\left[\begin{array}{c} y_{1}\\ y_{2}\\ \vdots \\ y_{n} \end{array}\right]$$

where *v_i_* indicates the tested value of a clinicopathological variable at day *i*, *v*_0_ represents a baseline measurement value, *x_i_* is a generated variable and *y_i_* is the corresponding score at day *i*. The generated matrix X and vector Y were then used for construction of the various machine learning models.

### Support Vector Machine Model

A support vector machine (SVM) is a category of generalized linear classifier that performs binary classification on data by supervised learning, having as a decision boundary the maximum margin hyperplane that solves the learning samples (Huang et al., 2018). SVM uses a hinge loss function to calculate the empirical risk and adds a regularization term to the solution system to optimize the structural risk. The model operates as a robust classifier using sparse data. SVM can perform nonlinear classification through kernel methods and represents a common kernel learning method. Using such methods, the robustness and sparsity of an SVM reduces the computational and memory overhead of the kernel matrices while ensuring that a reliable solution is obtained. The present study used the “SVR” function in the “scikit-learn” Python package to build the model. The parameter settings are: kernel=‘rbf’, degree=3, gamma=‘scale’, and C=1.0.

### Random Forest Model

A random forest (RF) refers to a classifier using multiple decision trees to train and predict samples. Output categories are determined by the mode of the output category of the individual trees (Blanchet et al., 2020). Random forests have the advantages of generating highly accurate classifiers while dealing with a large number of input variables and balancing errors. The present study used the “RandomForestRegressor” function in the “scikit-learn” Python package with all models constructed using the following parameters: n_estimators=100, criterion=‘squared_error’, min_samples_split=2, and min_samples_leaf=1.

### Multi-Layer Perceptron Model

A multi-layer perceptron (MLP) is a class of feedforward artificial neural network. Neural networks are operational models that consist of interconnections between a large number of nodes (or neurons). Each node represents a specific output function, described as an excitation function. The connection between every two nodes represents a weighted value for the signal passing through the connection, known as the weight, equivalent to the memory of the artificial neural network (Renganathan, 2019). The output of the network varies according to the method by which the network is connected, the weight value, and the excitation function. The network itself is generally an approximation of a certain algorithm or function in nature, and may also be an expression of a logic strategy. An MLP consists of at least three layers of nodes: an input layer, a hidden layer, and an output layer. Except for the input nodes, each node represents a neuron using a nonlinear activation function. The present study used the “MLPRegressor” function in the “scikit-learn” Python package, with parameter settings: solver=‘lbfgs’, alpha=1e-5, hidden_layer_sizes= (Li et al., 2010; Chen et al., 2015), and random_state=1.

### Statistical Analysis

Continuous variables (including age and baseline CD4 level) are presented as means ± standard deviation while the means of these variables in the 2 groups were compared using a student’s t-test. Categorical variables (sex, marital status, and HIV transmission route) are presented as numbers (or percentages of cases) while the prevalence of these variables was compared using a Pearson’s Chi-squared test. Due to the small sample sizes for a number of variables, comparisons were conducted using a Pearson’s Chi-squared test with Yates’ continuity correction. Pearson correlation analysis was used to calculate the pairwise correlation coefficients among all clinicopathological variables in the whole cohort. Pearson correlation analysis and univariate linear regression were used to explore the correlation between the original score and the predicted score generated by the three machine learning models. P-values < 0.05 were considered statistically significant. An independent sample t-test was used for statistical analysis of the biochemical indices and the viral load in each time group, for which 0.05 was the significance level.

## Results

### Population Characteristics

The population of patients was divided into two groups based on baseline CD4 concentrations (CD4 cell count < 200 cells/μl or ≥ 200 cells/μl). There were no differences in sex, age, or route of HIV transmission between the two groups. However, there was a significant difference in marital status between the two groups. The data indicated that the majority of patients with a CD4 cell count < 200 cells/μl were married, while a higher proportion of unmarried, divorced, and widowed patients were observed in the CD4 cell count ≥200 cells/μl (**Table 1**).

**Table 1:** Descriptive statistics stratified by baseline CD4 level.

	CD4 cell count < 200 cells/μl	CD4 cell count ≥ 200 cells/μl	*P* value^*^
Baseline CD4 level	86.29 ± 61.49	279.26 ± 49.44	<0.001
Sex			0.180
Male	42 (73.68)	19 (57.58)	
Female	15 (26.32)	14 (42.42)	
Age	40.55 ± 5.50	40.06 ± 11.70	0.819
Marital status			0.048
Unmarried	2 (3.39)	6 (16.67)	
Married	50 (84.75)	22 (61.11)	
Divorced	3 (5.08)	4 (11.11)	
Widowed	4 (6.78)	4 (11.11)	
HIV transmission			0.896
Heterosexual	53 (88.33)	31 (86.11)	
Intravenous drug use	2 (3.33)	1 (2.78)	
Unknown	5 (8.33)	4 (11.11)	

^*^Age and baseline CD4 level were compared using the Student’s t-test; sex, marital status, and HIV transmission route were compared using the Pearson’s Chi-squared test in the two groups.

### Comparison of Clinical Indicators and Viral Load in Each Group

As described above, all patients were categorized into a baseline CD4 cell concentration greater than or equal to 200, or less than 200 μl/ml. Depending on the follow-up period (from 0 to 9.8 years), the data were divided into 10 follow-up period groups (including the baseline group). All test indicators were compared between the two CD4 cell count groups at intervals of one year. It was found that there were significant differences in WBCs (P = 0.018), platelets (P = 0.001), ALT (P = 0.022), AST (P = 0.002), and hemoglobin (P = 0.002) between the two groups for follow-up periods of up to 4 years. There were significant differences in hemoglobin (P = 0.002) and AST (P = 0.002) between the two groups in the first year after diagnosis. For the follow-up period of 5 years, there was a significant difference in TC (P = 0.04) between the two groups, and a significant difference in creatinine (P = 0.014) for the 8 year follow-up (**Tables 2A**, **2B**).

**Table 2A:** Statistical analysis of clinicopathological variables in each time group.

	CD4	Baseline			0-1 year			1-2 years			3-4 years			4-5 years		
		N	Mean ± SD	p value	N	Mean ± SD	p value	N	Mean ± SD	p value	N	Mean ± SD	p value	N	Mean ± SD	p value
Viral load	≧200	14	32574.67 ± 54447.4	0.562	18	1683.06 ± 7134.36	0.491	25	845.36 ± 4195.21	0.321	23	34.04 ± 140.38	0.938	28	3.82 ± 20.22	0.782
	<200	19	58156.68 ± 156034.31		32	633.63 ± 3584.32		29	6739.34 ± 29093.25		37	30.51 ± 185.61		34	2.74 ± 9.5	
WBC	≧200	41	6.47 ± 6.69	0.121	68	5.36 ± 1.54	0.018*	62	5.4 ± 1.68	0.259	54	5.67 ± 1.47	0.118	49	6.07 ± 1.83	0.088
	<200	49	4.8 ± 3.03		114	4.7 ± 1.92		97	5.09 ± 1.67		78	5.27 ± 1.44		60	5.47 ± 1.82	
platelet	≧200	41	165 ± 59.56	0.244	68	190.62 ± 63.01	0.19	62	210.39 ± 59.54	0.001*	54	208.8 ± 72.1	0.05*	49	217.74 ± 77.37	0.066
	<200	49	147.04 ± 81.44		114	177.41 ± 67.02		97	178.04 ± 59.48		78	186.6 ± 56.71		60	192.75 ± 63.03	
creatinine	≧200	40	70.64 ± 24.19	0.734	63	65.65 ± 23.43	0.781	63	69.12 ± 17.66	0.678	53	69.06 ± 19.03	0.211	47	68.59 ± 21.59	0.207
	<200	48	72.31 ± 21.72		93	66.5 ± 14.55		93	70.43 ± 20.25		74	85.82 ± 95.5		57	73.88 ± 20.77	
TG	≧200	36	2.46 ± 4.33	0.639	61	1.87 ± 1.41	0.937	60	1.76 ± 0.91	0.389	53	3.17 ± 8.44	0.481	46	2.41 ± 2.33	0.549
	<200	39	3.33 ± 10.29		90	1.89 ± 1.55		85	1.93 ± 1.32		67	2.41 ± 2.31		55	2.14 ± 2.03	
ALT	≧200	40	32.44 ± 28.97	0.247	66	38.15 ± 32.98	0.555	62	27.55 ± 16.22	0.022*	53	32.13 ± 18.41	0.454	47	29.53 ± 15.5	0.531
	<200	49	41.94 ± 44.42		109	43.22 ± 64.64		95	38.03 ± 33.06		77	35.07 ± 24.09		55	31.73 ± 19.25	
Hb	≧200	41	146.98 ± 17.58	0.002*	66	146.82 ± 19.06	0.109	62	150.11 ± 16.48	0.723	54	155.54 ± 18.39	0.381	48	147.83 ± 28.5	0.333
	<200	46	130.24 ± 29.37		104	141.31 ± 23.26		89	151.15 ± 18.52		74	152.47 ± 20.29		57	172.26 ± 171.85	
TC	≧200	35	5.05 ± 6.11	0.299	61	4.41 ± 1.22	0.223	60	4.73 ± 0.81	0.534	53	4.56 ± 0.88	0.215	45	4.44 ± 1.01	0.139
	<200	39	3.98 ± 1.78		90	6.97 ± 16.29		84	5.21 ± 5.91		66	5.58 ± 5.87		55	4.76 ± 1.13	
AST	≧200	40	28.8 ± 9.49	0.002*	67	34.43 ± 21.03	0.288	63	28.04 ± 8.44	0.007*	55	30.44 ± 9.25	0.213	48	29.78 ± 14.89	0.284
	<200	49	45.04 ± 31.7		108	38.51 ± 26.69		91	38.77 ± 30.38		75	34.09 ± 20.09		57	35.52 ± 34.24	

The difference in bold is statistically significant. Symbol* represents a statistically different value.

**Table 2B:** Statistical analysis of clinicopathological variables in each time group.

	CD4	5-6 years			6-7 years			7-8 years			8-9 years			>=9 years		
		N	Mean ± SD	p value	N	Mean ± SD	p value	N	Mean ± SD	p value	N	Mean ± SD	p value	N	Mean ± SD	p value
Viral load	≧200	24	0	–	23	4.57 ± 16.46	0.369	12	2.42 ± 8.37	0.529	9	0	0.435	4	5.25 ± 10.5	0.356
	<200	25	0	–	24	1.29 ± 6.33		16	9.75 ± 39		14	4.64 ± 17.37		4	0	
WBC	≧200	44	5.67 ± 1.67	0.708	34	5.91 ± 1.45	0.664	17	6.74 ± 3.49	0.07	15	5.96 ± 1.45	0.736	6	6.16 ± 0.47	0.141
	<200	60	5.8 ± 1.98		46	5.75 ± 1.68		24	5.24 ± 1.56		18	5.75 ± 1.93		12	5.22 ± 1.43	
platelet	≧200	44	213.27 ± 72.74	0.128	34	218.79 ± 80.95	0.081	17	227.82 ± 58.97	0.056	15	216.53 ± 61.28	0.504	6	182 ± 59.04	0.797
	<200	60	192.87 ± 62.49		46	190.28 ± 63.44		24	182.25 ± 81.3		18	201.83 ± 62.81		12	175.25 ± 47.97	
creatinine	≧200	41	65.5 ± 15.93	0.134	34	69.22 ± 20.98	0.142	18	68.15 ± 16.55	0.066	15	70.1 ± 18.34	0.014*	6	96.17 ± 23.49	0.108
	<200	55	71.28 ± 20.23		44	75.51 ± 16.5		23	77.74 ± 15.76		18	83.56 ± 10.99		12	81.05 ± 14.46	
TG	≧200	42	1.98 ± 1.19	0.465	34	2.27 ± 3.54	0.636	18	2.56 ± 2.6	0.468	15	2.06 ± 1.46	0.713	6	2.5 ± 1.82	0.061
	<200	51	2.26 ± 2.19		42	1.99 ± 1.5		22	5.99 ± 19.68		18	1.9 ± 0.98		11	1.36 ± 0.39	
ALT	≧200	42	31.59 ± 27.39	0.633	32	24.53 ± 26.07	0.459	17	22.96 ± 9.6	0.135	14	21.86 ± 10.38	0.107	5	20.48 ± 6.57	0.696
	<200	56	29.52 ± 14.88		44	28.03 ± 14.68		23	28.79 ± 13.35		17	30.92 ± 18.01		12	23.01 ± 13.34	
Hb	≧200	44	152.17 ± 21.74	0.826	34	150.08 ± 21.9	0.421	17	152.24 ± 22.54	0.824	15	154.27 ± 19.34	0.994	6	156 ± 9.98	0.573
	<200	53	153.02 ± 16.07		43	153.63 ± 16.55		21	150.81 ± 16.7		16	154.31 ± 15.43		10	150.6 ± 21.31	
TC	≧200	42	4.43 ± 1.01	0.04*	34	4.8 ± 1.65	0.856	18	4.41 ± 0.88	0.802	15	4.34 ± 0.8	0.08	6	4.25 ± 1.03	0.251
	<200	51	4.89 ± 1.09		41	4.74 ± 0.95		21	4.51 ± 1.41		18	4.78 ± 0.59		11	4.79 ± 0.8	
AST	≧200	43	31.72 ± 13.84	0.713	34	23.97 ± 7.51	0.077	18	22 ± 11.61	0.083	15	22.67 ± 5.98	0.131	6	21.78 ± 3.08	0.346
	<200	56	30.48 ± 18.31		44	30.44 ± 19.98		23	34.53 ± 27.98		17	27.26 ± 9.96		12	25.43 ± 8.8	

*Means p value < 0.05.

All variables in the two groups were compared using the Student’s t-test. The difference in bold is statistically significant.

### Correlation of Clinical Indices

**Figure 1:**
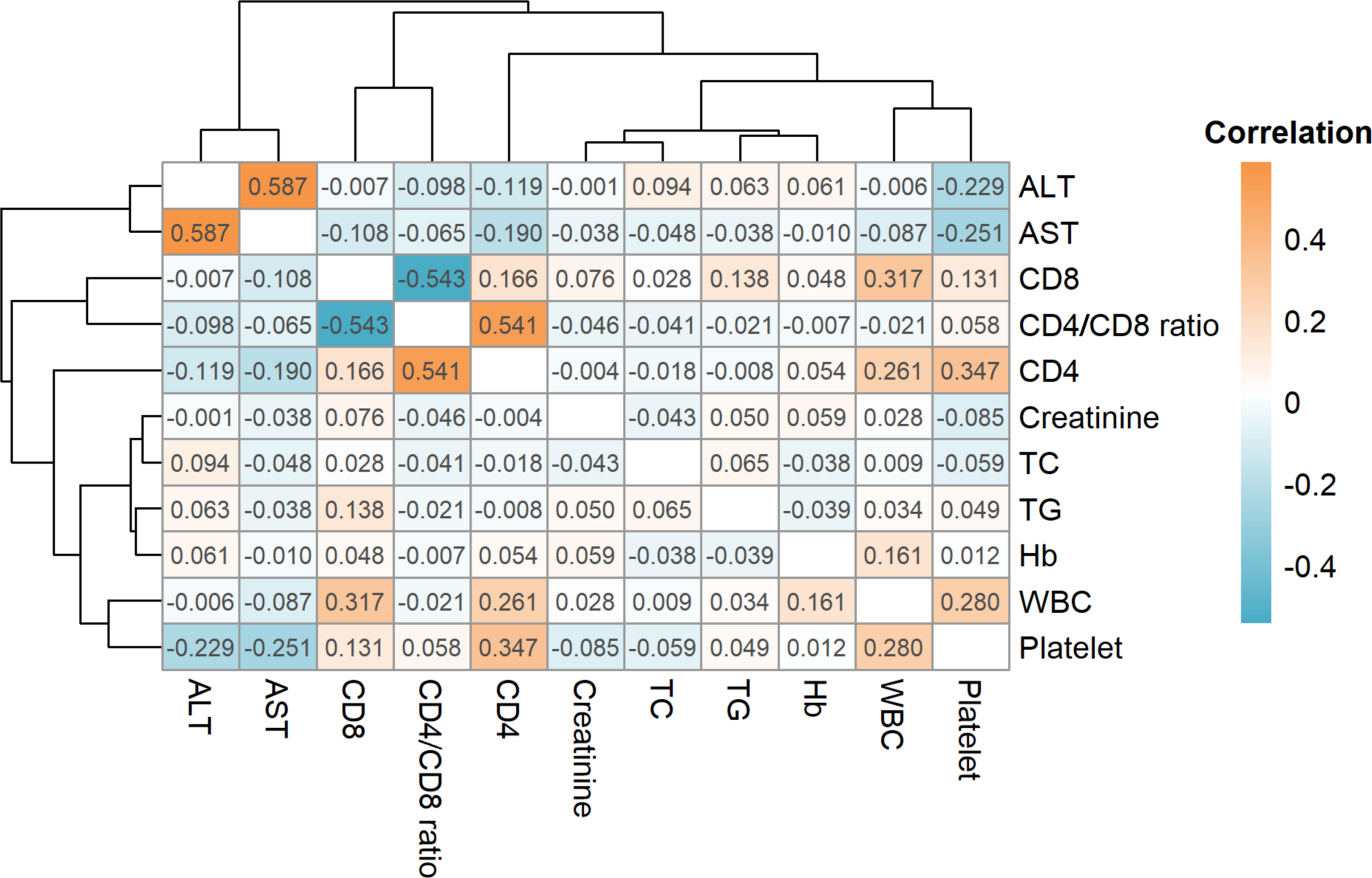
Correlations for all clinicopathological variables. The orange color indicates a positive correlation while cyan indicates a negative correlation. ALT, alanine aminotransferase; AST, aspartate aminotransferase; CD4, CD4^+^; T cell, CD8: CD8^+^ T cell; TC, total cholesterol; TG, total cholesterol; Hb, total cholesterol; WBC, white blood cell.

The results indicate that the ALT and AST levels demonstrated a strong positive correlation (r = 0.587) and the CD4 level was also strongly positively correlated with the CD4/CD8 ratio (r = 0.541), whereas the CD8 level was strongly negatively correlated with the CD4/CD8 ratio (r = -0.543, **Figure 1**). However, the CD4 level was only weakly positively correlated with the CD8 level (r = 0.166). Furthermore, CD4 was positively correlated with WBCs (r = 0.261) and platelets (r = 0.347) while CD8 was positively correlated with WBCs (r = 0.317). Platelets were negatively correlated with ALT (r = -0.229) and AST (r = -0.251), and positively correlated with WBCs (r = 0.280).

### Predictive Performance of the Three Machine Learning Models

In patients with a CD4 cell count of < 200 cells/μl, there were significant correlations between the predicted results of the SVM model and the patient data for CD4 (r = 0.390, P = 0.045), CD4/CD8 ratio (r = 0.721, P < 0.001), platelets (r = 0.435, P = 0.022), TG (r = 0.614, P = 0.005), and AST (r = 0.569, P = 0.012). Additionally, the predicted results of the RF model were significantly correlated with the patient data for CD8 (r = 0.368, P = 0.028), CD4/CD8 ratio (r = 0.662, P = 0.002), platelets (r = 0.563, P = 0.013), and TG (r = 0.536, P = 0.008). Finally, the predicted results of the MLP model were significantly correlated with the patient data for CD8 (r = 0.412, P = 0.008), CD4/CD8 ratio (r = 0.554, P = 0.015), and platelets (r = 0.451, P = 0.016). For the CD4 cell count ≥ 200 cells/μl group, a significant correlation was observed for data predicted by the SVM model and the patient data for CD4 (r = 0.365, P = 0.036), CD4/CD8 ratio (r = 0.807, P < 0.001), WBCs (r = 0.577, P = 0.005), TC (r = 0.482, P = 0.011), and ALT (r = 0.362, P = 0.035). The results predicted by the RF model were significantly correlated with the patient data for CD4 (r = 0.513, P = 0.002), CD8 (r = 0.634, P = 0.003), CD4/CD8 ratio (r = 0.898, P < 0.001), WBCs (r = 0.452, P = 0.008), and platelets (r = 0.484, P = 0.004), while there were significant correlations for the MLP model for CD4 (r = 0.356, P = 0.028), CD8 (r = 0.315, P = 0.032), and CD4/CD8 ratio (r = 0.837, P < 0.001). The results above demonstrate that the three machine learning models exhibited a superior predictive performance in patients with a CD4 cell count ≥ 200 cells/μl than in those with a CD4 cell count < 200 cells/μl (**Table 3**).

**Table 3:** Predictive performance of the three machine learning models for clinicopathological variables.

Variables	SVM model		RF model		MLP model	
	R	P	R	P	R	P
CD4 cell count < 200 cells/μl						
CD4	0.390	0.045	0.219	0.185	0.257	0.207
CD8	0.025	0.241	0.368	0.028	0.412	0.008
CD4/CD8 ratio	0.721	< 0.001	0.662	0.002	0.554	0.015
WBC	0.293	0.200	0.293	0.243	0.297	0.116
Platelets	0.435	0.022	0.563	0.013	0.451	0.016
Creatinine	0.136	0.205	0.069	0.225	0.206	0.177
TC	0.219	0.105	0.112	0.403	0.126	0.431
TG	0.614	0.005	0.536	0.008	0.098	0.599
ALT	0.237	0.101	0.359	0.057	0.105	0.859
AST	0.569	0.012	0.082	0.442	0.271	0.064
Hb	0.031	0.728	0.042	0.543	0.073	0.652
CD4 cell count ≥ 200 cells/μl						
CD4	0.365	0.036	0.513	0.002	0.356	0.028
CD8	0.190	0.108	0.634	0.003	0.315	0.032
CD4/CD8 ratio	0.807	< 0.001	0.898	< 0.001	0.837	< 0.001
WBC	0.577	0.005	0.452	0.008	0.383	0.068
Platelets	0.290	0.231	0.484	0.004	0.213	0.185
Creatinine	0.091	0.381	0.170	0.386	0.068	0.827
TC	0.482	0.011	0.341	0.052	0.261	0.127
TG	0.191	0.279	0.078	0.558	0.058	0.691
ALT	0.362	0.035	0.320	0.065	0.082	0.541
AST	0.298	0.161	0.277	0.191	0.183	0.241
Hb	0.120	0.381	0.254	0.232	0.081	0.391

Pearson correlation statistics are displayed as correlation coefficients (R) and P values.

### Predictions of the CD4/CD8 Ratio

**Figure 2:**
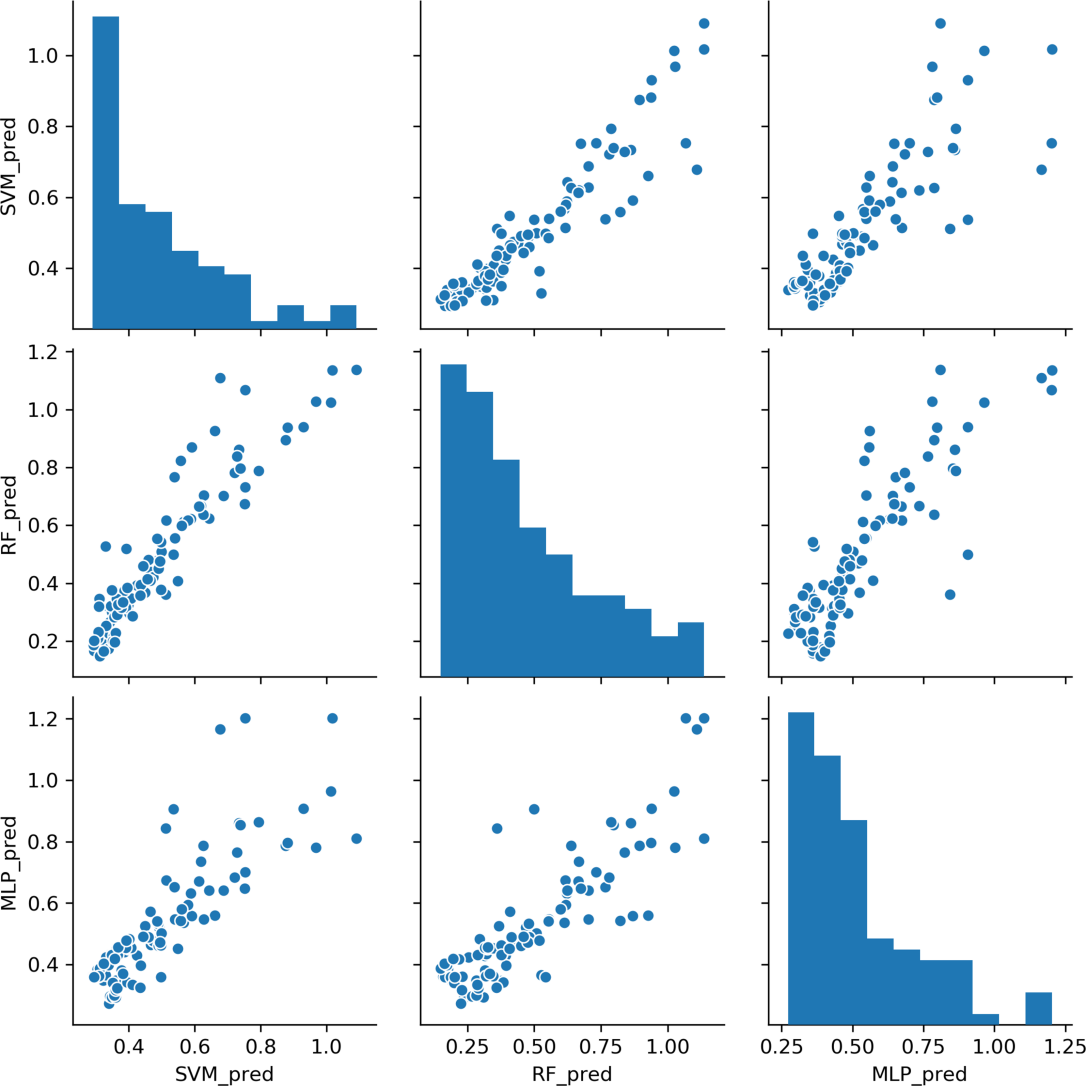
Comparisons of CD4/CD8 ratio predictions for the three machine learning models. Each point represents a sample. SVM, support vector machine; RF, random forest; MLP, multi-layer perceptron.

**Figure 3:**
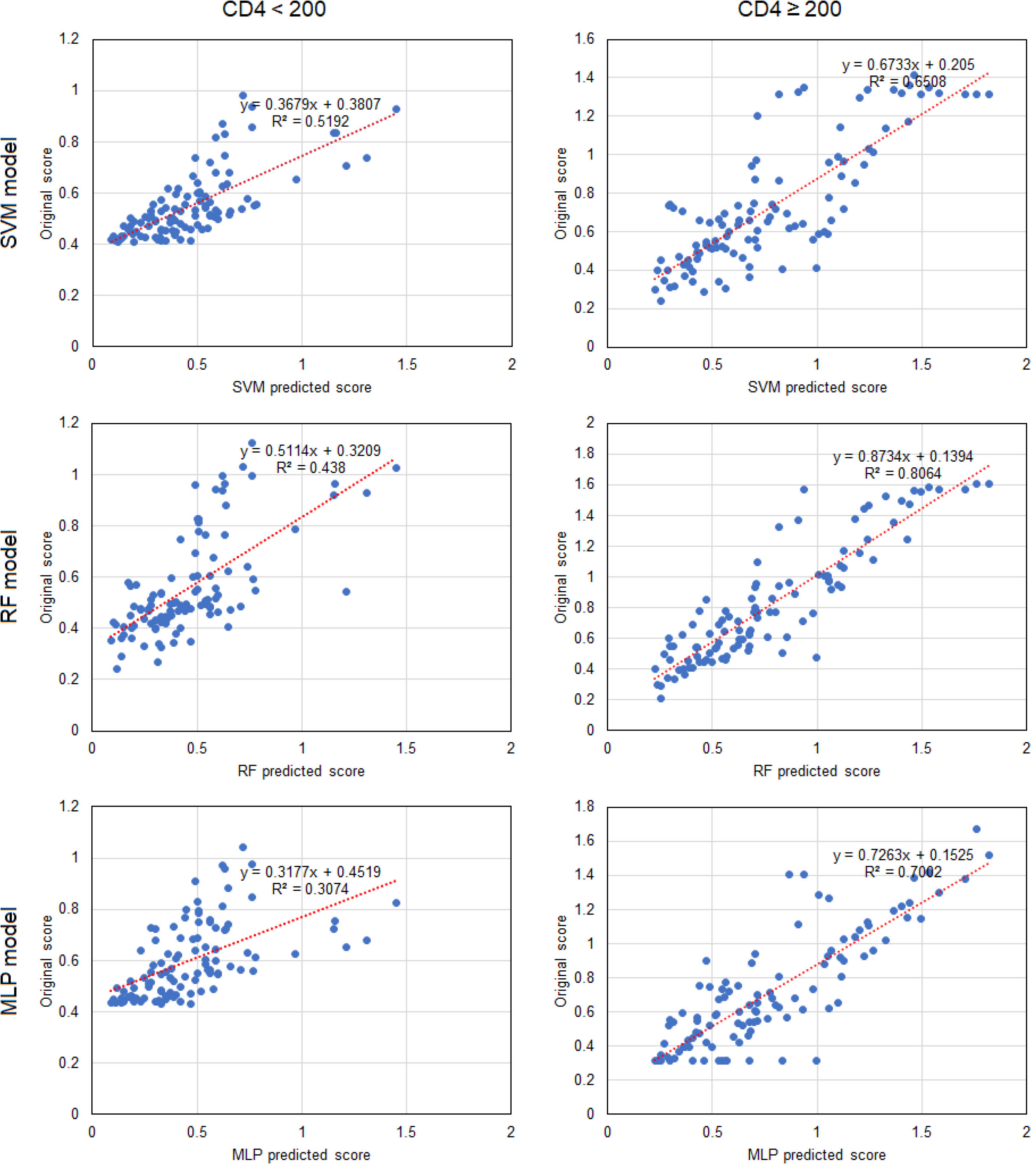
Linear correlations of predicted scores and patient data for CD4/CD8 ratio in the three machine learning models. Each point represents a sample. CD4, CD4^+^ T cell; SVM, support vector machine; RF, random forest; MLP, multi-layer perceptron.

Based on the results above, we found that the best predictive performance for CD4/CD8 ratio was achieved by the machine learning model. All three models demonstrated highly consistent predictions (**Figure 2**). In patients with a CD4 cell count of < 200 cells/μl, the SVM model displayed the best predictive performance (r^2^ = 0.519), followed by the RF model (r^2^ = 0.438), with the MLP model (r^2^ = 0.307) found to be worst. In patients with a CD4 cell count of ≥ 200 cells/μl, the RF model exhibited the best predictive performance (r^2^ = 0.806), followed by the MLP model (R^2^ = 0.700), with the SVM model found to be worst (r^2^ = 0.651). The results indicate that it may be appropriate to utilize the SVM model to predict the CD4/CD8 ratio for patients with a CD4 cell count < 200 cells/μl, and the RF model for those with a CD4 cell count of ≥ 200 cells/μl (**Figure 3**).

## Discussion

The number and function of lymphocytes are directly related to immune system function. The CD4 cell count is among the most critical indicators of immune function, lower counts indicating weaker immune system function (Frontiers in Cellular and Infection Microbiology, 2018). However, not all T-cell subsets become attenuated. CD4^+^ T cells are helper lymphocytes that secrete cytokines that activate other immune cells. CD8^+^ T lymphocytes, also known as cytotoxic T cells, directly destroy virus-infected and tumor cells. Following HIV infection, the synthesis of CD4^+^ T cells is reduced and their destruction increases causing their number to progressively decrease, although the number of CD8^+^ T cells increases significantly and they become functionally activated (Masiá et al., 2016). Therefore, while observing the destruction of immune function in patients following HIV infection, or its reconstruction, in addition to an intuitive index of plasma viral load, attention should also be paid to the number of CD4^+^ T cells, the absolute number of CD8^+^ T cells, the CD4/CD8 ratio, and other immune activation parameters (Cohen Stuart et al., 2000). The CD4/CD8 ratio is often described as a marker of immune status in the general population and is of interest in studies of HIV-infected individuals. There is increasing evidence that it can be used to identify HIV-infected individuals with persistent immune dysfunction who, despite having a normal CD4 count during treatment, have a higher risk of non-AIDS events and death. It has been confirmed that early administration of ART is related to a rapid increase in CD4^+^ count and the CD4/CD8 ratio (Rajasuriar et al., 2015), a direct indicator of immune function reconstruction. A decrease in the CD4/CD8 ratio is generally associated with an increase in morbidity and mortality in HIV-unrelated diseases (Mussini et al., 2015).

In the present study, correlation analysis of the clinical information of patients with CD4^+^ T cell number demonstrated that there was no statistical difference in gender, age, or route transmission of HIV (heterosexual transmission, intravenous drug use, or unknown) with baseline CD4 cell count while marital status was statistically correlated. A large number of HIV/AIDS patients in the married group had a CD4 cell count < 200 cells/μl, while the majority that were unmarried, divorced, or widowed had ≥ 200 cells/μl. A possible reason may be due to cultural factors and a sense of fear towards divulging their HIV/AIDS status, instead intentionally concealing it to prevent the other party knowing their infection status. Even when symptoms appear, a delay in seeking medical treatment would delay the diagnosis until the onset of impaired immune function.

Correlation analysis of the clinical characteristics demonstrated that ALT levels were positively correlated with AST levels (R = 0.587), while CD4 levels were positively correlated with the CD4/CD8 ratio (R = 0.541). In addition, CD4 was positively correlated with platelet count (R = 0.347). Platelet count was negatively correlated with ALT (r = -0.229) and AST (R = -0.251), and positively correlated with WBCs (r = 0.280). A number of previous studies (Chen et al., 2007; Gupta et al., 2007) have stated that the combination of total lymphocyte count, hemoglobin, and platelet count improves the accuracy of CD4 count prediction. In addition, clinical practice suggests that the number of CD4^+^ T lymphocytes may also be related to red blood cell and white blood cell count, similar to observations in the present study.

Since the HIV/AIDS patients were treated with ART following diagnosis, they experienced reconstruction of the immune system and gradual recovery of immune function over 3-4 years of follow-up. The duration of the reconstruction period was dependent on patient characteristics. Therefore, indicators of disease in the patients during the reconstruction period were not stable. Fluctuation caused clear differences between the two groups. However, after 4 years of follow-up, all aspects of the monitored indicators tended to stabilize in the patients who had achieved immune reestablishment, removing any significant difference between the two groups. Differences in total cholesterol and creatinine between the two groups in the late follow-up period may have been caused by side effects or complications of the drugs or damage to organ function caused by the antiviral drugs.

In the present study, the three machine learning models, namely SVM, RF and MLP, were constructed by simultaneously incorporating the clinical indicators of HIV/AIDS patients, such as routine blood tests, lymphoid subgroup count, viral load, liver and renal function, and blood lipids. Baseline values and follow-up monitoring indicators were used to analyze and predict the possible test results of these indicators during treatment and follow-up testing. In both groups of baseline CD4 cell counts, the CD4/CD8 ratio predicted by the three models was significantly correlated with the patient data. In patients with a CD4 cell count of < 200 cells/μl, only the predicted platelet count for the three models was correlated with the patient data. In conclusion, the predictive capability of the three machine learning models was superior in patients with a CD4 cell count of ≥200 cells/μl rather than < 200 cells/μl.

SVM is considered among the most accurate methods of all the well-known data mining algorithms. It is a novel small-sample learning method with a solid theoretical foundation. RF can process high dimensional data, the training speed is fast, and it easily obtains the extent of the importance of different features. MLP, as a neural network machine learning method, can achieve better predictive performance following a training and learning process. In general, machine learning models have different predictive performance regarding CD4 counts, as observed for the different machine learning models in the present study, with the CD4/CD8 ratio providing the best performance, for which all three models were highly consistent. Specifically in patients with a CD4 cell count of ≥200 cells/μl, the RF model demonstrated the best predictive performance (r^2^ = 0.806), including for predicting the CD4/CD8 ratio, whereas the SVM model should be used to predict the CD4/CD8 ratio in patients with a CD4 cell count of < 200 cells/μl. Of course, the RF model would theoretically demonstrate superior predictive performance if the sample size for machine learning was larger.

After treatment with ART, the slow increase in CD4^+^ cell count is likely to lead to immune reconstitution. The high baseline CD4/CD8 ratio was associated with successful immune reconstitution, in accordance with previous studies that have demonstrated the critical role of CD4/CD8 ratio normalization (Serrano-Villar et al., 2013; Mussini et al., 2015). Therefore, the number of CD4 cells and the CD4/CD8 ratio after treatment in HIV/AIDS patients are key factors for successful treatment.

The present study was influenced by cases of loss and, consequently, the sample size for long-term follow-up was relatively small. The predictive role of baseline CD4^+^ cell count and the CD4/CD8 ratio in immune reconstitution has become clearer as the sample size has continuously increased. The additional detection of CD4^+^ T cell subsets in enrolled cases in the future will yield more effective evidence, as there is increasing evidence that different subtypes of CD4^+^ cells influence immune reconstitution (Funderburg et al., 2013; Lu et al., 2016) and that a baseline percentage of naive CD4^+^ T cells is a good prognostic factor for immune reconstitution during long-term treatment (Guo et al., 2016). Furthermore, it is possible to optimize prediction through the integration of models. Therefore, we will further analyze the relationship between changes in the monitoring indicators included here and changes in CD4 count, thereby allowing prediction of changes in CD4 count through the use of data mining methods. These will be considered in additional research.

## Conclusion

In the present study, three machine learning models, namely SVM, RF and MLP, were constructed by including clinical monitoring indicators such as routine blood examination, lymphocyte subset counts, viral load, liver and renal function, and blood lipid levels in HIV/AIDS patients at baseline and the follow-up period. Baseline and follow-up results were used to analyze and predict the outcome of these measures after treatment and follow-up testing. The results demonstrated that the predictive capability of the three models was better for the group with a CD4 cell count ≥200 cells/μl than for patients with < 200 cells/μl. For both groups, the three models yielded the best predictive performance for the CD4/CD8 ratio, for which the results were highly consistent. In patients with a CD4 cell count of < 200 cells/μl, the SVM model exhibited the best performance for predicting the CD4/CD8 ratio, while in patients with a CD4 cell count of ≥200 cells/μl, the RF model was best.

## Data Availability Statement

The original contributions presented in the study are included in the article/supplementary material. Further inquiries can be directed to the corresponding authors.

## Ethics Statement

The research protocol used in the present study has been reviewed by the Ethics Committee of the First Affiliated Hospital of Kunming Medical University. Informed consent was obtained from all participants included in the study prior to enrollment, and all information and data were confirmed for analysis.

## Author Contributions

BL, MS, Y-QK, YL, and DL conceived and designed the study. BL, ML, Y-QK, XW, and RZ collected the reagents and study materials. BL, ML, YS, XL, XW, and RZ performed the laboratory experiments. BL, RZ, MS, YL, and DL analyzed the data. BL, ML, MS, YL, and DL wrote and revised the manuscript. All authors approved the final manuscript.

## Funding

The study was supported by the Special Funds for Yunnan Province Medical academic leader (D-2018025), a Scientific Research Fund project of Yunnan Education Department (2020J0171), High-level Healthy Talents of Yunnan Province (D-2019022), Natural Science Foundation of Yunnan Province (202101AU070124), and open projects of Yunnan Key Laboratory of Laboratory Medicine (JYZDSYS202004 & JYZDSYS202101).

## Conflict of Interest

The authors declare that the research was conducted in the absence of any commercial or financial relationships that could be construed as a potential conflict of interest.

## Publisher’s Note

All claims expressed in this article are solely those of the authors and do not necessarily represent those of their affiliated organizations, or those of the publisher, the editors and the reviewers. Any product that may be evaluated in this article, or claim that may be made by its manufacturer, is not guaranteed or endorsed by the publisher.

## Abbreviations

SVM, Support vector machine; RF, Random forest; MLP, Multi-layer perceptron.
